# Identification of rare thalassemia variants using third-generation sequencing

**DOI:** 10.3389/fgene.2022.1076035

**Published:** 2023-01-04

**Authors:** Qin Liu, Qianting Chen, Zonglei Zhang, Shiyi Peng, Jing Liu, Jialun Pang, Zhengjun Jia, Hui Xi, Jiaqi Li, Libao Chen, Yinyin Liu, Ying Peng

**Affiliations:** ^1^ Hunan Provincial Maternal and Child Health Care Hospital, Changsha, China; ^2^ Berry Genomics Corporation, Beijing, China

**Keywords:** thalassemia, third-generation sequencing, genetic analysis, hemoglobin, variant

## Abstract

Routine PCR, Sanger sequencing, and specially designed GAP-PCR are often used in the genetic analysis of thalassemia, but all these methods have limitations. In this study, we evaluated a new third-generation sequencing-based approach termed comprehensive analysis of thalassemia alleles (CATSA) in subjects with no variants identified by routine PCR, Sanger sequencing, and specially designed GAP-PCR. Hemoglobin testing and routine PCR tests for 23 common variants were performed for 3,033 subjects. Then, Sanger sequencing and specially designed GAP-PCR were performed for a subject with no variants identified by routine PCR, no iron deficiency, and positive hemoglobin testing. Finally, the new CATSA method was conducted for the subjects with no variants identified by Sanger sequencing and specially designed GAP-PCR. In the 49 subjects tested by CATSA, eight subjects had variants identified. Sanger sequencing and independent PCR confirmed the CATSA result. In addition, it is the first time that Hb Lepore was identified in Hunan Province. In total, traditional methods identified variants in 759 of the 3,033 subjects, while CATSA identified additional variants in eight subjects. CATSA showed great advantages compared to the other genetic testing methods.

## 1 Introduction

Thalassemia is the most widely distributed monogenic autosomal recessive disorder in the world and is caused by variants in human globin genes (Higgs et al., 2012; Williams and Weatherall, 2012; Taher et al., 2018). The most common types of thalassemia are α-thalassemia (OMIM: #604131) and β-thalassemia (OMIM: #613985), which are caused by variants in the α-globin genes and β-globin genes, respectively. Phenotypes of thalassemia range from asymptomatic to severe (even lethal) anemia, and these phenotypes are usually well correlated with the number of mutated globin genes (Modell and Darlison, 2008; Jiang et al., 2022; Ning et al., 2022; Zhong et al., 2022). There is no effective treatment for thalassemia, and carrier screening and prenatal diagnosis for high-risk couples are the main measures of the thalassemia control plan (Goonasekera et al., 2018). According to the Guidelines of Antenatal Thalassemia Screening, the screening modalities include hematological testing and hemoglobin analysis. Genetic testing is then conducted for those with phenotypic traits associated with thalassemia (Ryan et al., 2010).

The genotypes of thalassemia are region-specific, and 23 most common variants in HBA and HBB genes are routinely screened by PCR in diagnostic laboratories for thalassemia testing in China (Xu et al., 2004). However, more and more rare variants ranging from single-base changes to large rearrangements have been reported, suggesting the necessity to include these relative rare variants in the genetic testing (Cao and Galanello, 2010; Galanello and Cao, 2011). For example, α-thalassemia usually results from NC_000016.10:g.165401_184701del (--^SEA^), NG_000006.1:g.34247_38050del (-α^3.7^), NC_000016.10:g.169818_174075del (-α^4.2^), HBA2:c.427T>C (Hb Constant Spring), HBA2:c.377T>C (Hb Quong Sze), and HBA2:c.369C>G (Hb Westmead) that mainly impair the coding regions of α-globin genes. Recently, it was found that large deletions in the enhancer of α-globin genes such as NC_000016.10:g.113161_113902del ((αα)^JX^) can also cause α-thalassemia (Wu et al., 2017). Variants in the δ-globin gene (HBD) will not cause thalassemia, but they can lead to a decrease in Hb A2. Thus, the increase in Hb A2 caused by β-thalassemia may be impaired by HBD variants (Liu et al., 2013).

GAP-polymerase chain reaction (GAP-PCR) and Sanger sequencing are often used in the genetic analysis of rare thalassemia variants. However, these methods all have limitations (Brancaleoni et al., 2016; Traeger-Synodinos and Harteveld, 2017). Recently, third-generation sequencing (TGS) has been emerging as a new technique in genetic testing for thalassemia. Compared to PCR and Sanger sequencing, TGS has the advantages of high-throughput long reads and no amplification during the sequencing process, enabling highly accurate identification of repeat GC-rich and highly homologous regions. In addition, TGS can detect both known and unknown variants and determine whether two or more variants are in cis- or trans-configurations (Ardui et al., 2018; Liu et al., 2022). The clinical utility of a third-generation sequencing-based approach termed comprehensive analysis of thalassemia alleles (CATSA) in carrier screening of thalassemia was vigorously validated in blind clinical studies with a large cohort of samples, and CATSA demonstrated great advantages in terms of detecting range and accuracy compared to routine PCR and multiple ligation-dependent probe amplification (Liang et al., 2021; Luo et al., 2022; Zhuang et al., 2022). In this study, the previous CATSA method was modified to cover more thalassemia variants. Hemoglobin testing and routine PCR for 23 common variants were performed for 3,033 subjects. Then, Sanger sequencing and specially designed GAP-PCR were performed for the subject with no variants identified by routine PCR, with no iron deficiency, and who was positive for hemoglobin testing. Then, the new CATSA method was conducted for the subjects with no thalassemia variants identified to see whether it can detect the variants missed by PCR and Sanger sequencing.

## 2 Materials and methods

### 2.1 Sample enrollment

Subjects in this retrospective study were recruited at Hunan Provincial Maternal and Child Health Care Hospital during October 2020–December 2021. The proportion of adults and children was 95% and 5%, respectively. The enrolled samples were all single patients. Since silent carriers showed negative results for hemoglobin testing, the screening program also involved performing routine PCR for individuals who tested negative for hemoglobin testing. Hemoglobin testing and routine PCR were performed simultaneously for the 3,033 individuals. Study ethics approval was granted by the hospital review board, and all the subjects or their legal guardians signed informed written consent.

### 2.2 Hemoglobin testing

Hemoglobin testing was carried out by standard blood assays and hemoglobin electrophoresis. Profiling for standard blood assays included mean corpuscular volume (MCV) and mean corpuscular hemoglobin (MCH). Parameters measured in hemoglobin electrophoresis included hemoglobin A2 (Hb A2), hemoglobin A (Hb A), and hemoglobin F (Hb F). Hemoglobin testing negative was defined as MCV ≥ 80 fL, MCH ≥ 27 pg, Hb A2 levels between 2.5% and 3.5%, and Hb F ≤ 5%. Otherwise, they were defined as hemoglobin testing positive.

### 2.3 Genetic analysis by PCR and Sanger sequencing

Routine PCR including GAP-PCR and PCR-reverse dot blot hybridization (RDB) was conducted according to the manufacturer’s protocol (Yaneng, Shenzhen, China) at Hunan Provincial Maternal and Child Health Care Hospital to detect 23 common thalassemia variants. Variants tested by GAP-PCR included --^SEA^ (Southeast Asia), -α^3.7^ (rightward), and -α^4.2^ (leftward). Variants tested by PCR-RDB included HBA2:c.427T>C, HBA2:c.377T>C and HBA2:c.369C>G, HBB:c.316–197C>T (IVS II-654C>T), HBB:c.126_129delCTTT (CD 41/42(-CTTT)), HBB:c.52A>T (CD17 AAG>TAG), HBB:c.216_217insA (CD71/72(+A)), HBB:c.-78A>G (-28(A>G)), HBB:c.-79A>G (-29(A>G)), HBB:c.-82C>A (-32 (C>A)), HBB:c.79G>A (CD 26 GAG>AAG), HBB:c.-11_-8delAAAC (CAP +40 to +43 (-AAAC)), HBB:c.84_85insC (CD 27/28(+C)), HBB:c.130G>T (CD 43 (GAG>TAG)), HBB:c.94delC (CD 31 (-C)), HBB:c.-80 (T>C) (-30 (T>C)), HBB:c.45_46insG (CD 14/15 (+G)), HBB:c.2T>G (Init CD ATG>AGG), HBB:c.92+1G>T (IVS I-1 (G>T)), and HBB:c.92+5G>C (IVS I-5 (G>C)). GAP-PCR was also performed to detect six rare thalassemia variants, namely, NG_000006.1:g.10664_44164del33501 (--^THAI^), NG_000006.1:g.[14373_36299del21927; insGGGAAGGGTGGGTGGGAATAACAGCTTTT] (-α^21.9^), NG_000006.1:g.9079_36718del27640 (-α^27.6^), NG_000007.3:g.69997_71353del1357 (Taiwanese), NC_000011.10:g.5169918_5248821del (Chinese ^G^γ(^A^γδβ)^0^), and NC_000011.10:g.5201647_5229059del (SEA-HPFH). Sanger sequencing was used to detect rare single nucleotide variants (SNVs) in *HBA1*, *HBA2*, and *HBB* genes.

### 2.4 Genetic analysis by CATSA

Samples were sent to Berry Genomics for CATSA sequencing. Previous multiplex long PCR analysis (Liang et al., 2021) was modified to amplify additional genomic regions including HBD and HS40 regions. The region analyzed for HS40 was chr16: 109217–117302. The region analyzed for HBA1 and HBA2 was chr16: 169296–178914. The region analyzed for HBB and HBD was chr11: 5223675–5235660. Then, a barcoded adaptor was ligated to the PCR products by a one-step end-repair and ligation reaction. Unligated products were removed by exonucleases (Enzymatics), and pre-libraries were pooled together by equal mass. Next, the SMRT bell library was generated from the pooled library using Sequel Binding and Internal Ctrl Kit 3.0 (Pacific Biosciences) and sequenced using the CCS mode on the Sequel II platform (Pacific Biosciences). The raw subreads were converted to CCS reads by CCS software (Pacific Biosciences) and demultiplexed by barcodes using lima in the Pbbioconda package (Pacific Biosciences). The demultiplexed CCS reads were then aligned to genome build hg38 with pbmn2. FreeBayes1.3.4 (https://www.geneious.com/plugins/freebayes; Biomatters, Inc., San Diego, CA) was used to call SNVs and indels. Structural variations were called based on HbVar, Ithanet, and LOVD databases.

### 2.5 Confirmation of variants detected by CATSA

(αα)^JX^, NG_000006.1:g.(31695_31724)_(42846_42867)del (--^11.1^), and NG_000007.3: g.63632_71046del (Hb Lepore) identified by CATSA were confirmed by GAP-PCR. HBB:c.315+308delA (IVS II-308 (-A)), HBB:c.315+337A>G (IVS II-337A>G), and HBD:c.127T>C (CD 42 TTT>CTT [Phe>Leu]) were verified by Sanger sequencing.

## 3 Results

### 3.1 Thalassemia screening and summary of the CATSA results

**FIGURE 1:**
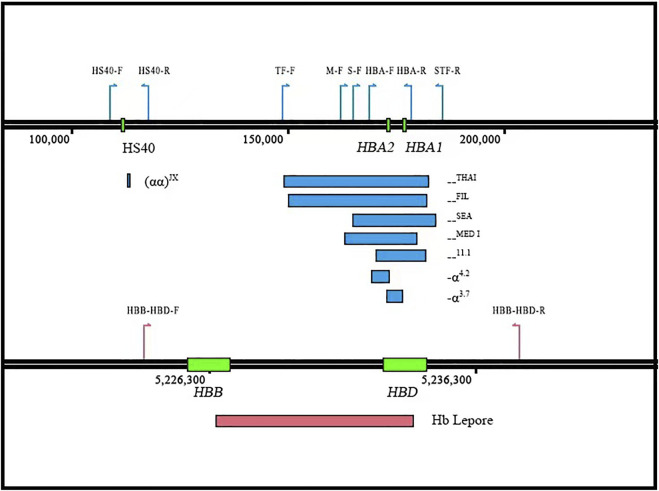
Design of the new CATSA method. Three sets of primer pairs were designed to individually detect the --^THAI^ and --^FIL^ (TF-F/STF-R), the --^SEA^ (S-F/STF-R), the --^11.1^ (HBA-F/STR-R), and the --^MED^-I (M-F/STR-R) deletions. A fourth primer pair was designed to detect smaller deletions involving HBA1 and HBA2 (-α^3.7^), HBA2 deletions (-α^4.2^), and HBA1 and HBA2 SNVs/indels as well as wild-type sequences (HBA-F/HBA-R). A fifth primer pair (HS40-F/R) was designed to amplify the (αα)^JX^/αα. A sixth primer pair (HBB-HBD-F/R) was designed to amplify the entire HBB and HBD genes to detect known SVs, SNVs, and indels. The position of the different primers is indicated by colored arrows.

**FIGURE 2:**
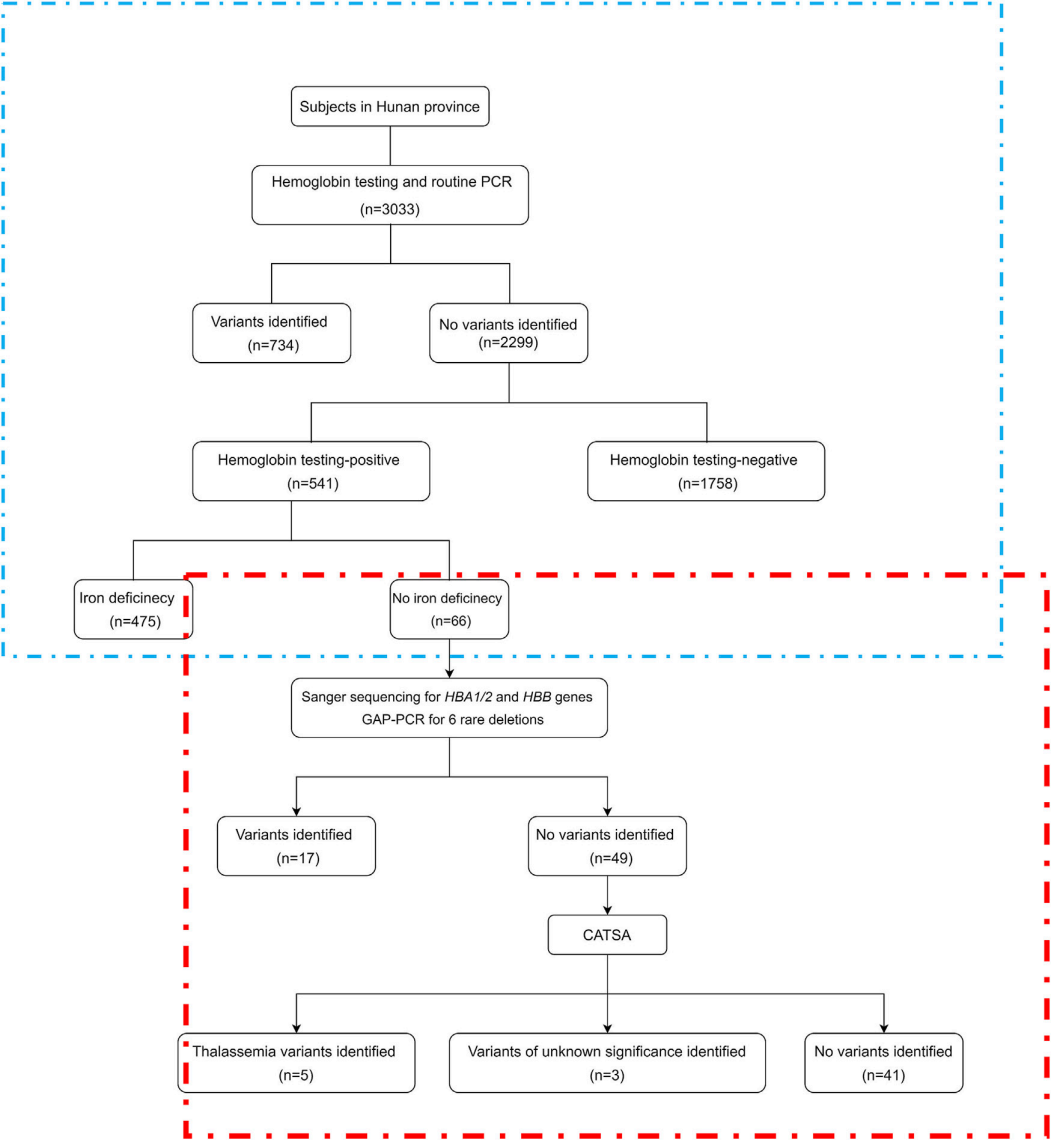
Study flowchart. CATSA, a third-generation sequencing approach termed comprehensive analysis of thalassemia alleles. PCR, polymerase chain reaction. The part in the blue box is the retrospective analysis, and the part in the red box is the study flowchart.

To detect more thalassemia variants, the previous CATSA method was modified to incorporate new primers (Figure 1). Then, hemoglobin testing and routine PCR for 23 common variants were performed for 3,033 subjects in Hunan Province. From the tests performed, thalassemia variants were identified in 734 subjects, and 2,299 subjects had no thalassemia variants identified in their genomes. Among the 2,299 subjects, 1,758 were hemoglobin testing negative and 541 were hemoglobin testing positive. Among the 541 hemoglobin testing-positive subjects, 475 had iron deficiency and 66 had no iron deficiency. Next, Sanger sequencing for *HBA1/2* and *HBB* genes and specially designed GAP-PCR for six rare deletions including --^THAI^, -α^21.9^, -α^27.6^, Taiwanese, Chinese ^G^γ(^A^γδβ)^0^, and SEA-HPFH were carried out for the 66 subjects, and 17 subjects were identified with thalassemia variants. Then, CATSA was conducted for the 49 subjects with no thalassemia variants identified by Sanger sequencing and specially designed GAP-PCR (Figure 2). In the 49 subjects tested by CATSA (Supplemental Table S1), eight subjects had variants identified. Among them, three presented α^0^ variants, one presented the Hb Lepore variant, three presented variants of potential pathogenicity, and one presented a *HBD* variant (Table 1). In total, traditional methods identified variants in 759 of the 3,033 subjects recruited in Hunan Province, while CATSA identified additional variants in eight subjects.

**TABLE 1:** Variants identified in the samples by CATSA.

Subject	MCV (fL)	MCH (pg)	HbA2 (%)	Hb F (%)	HBA variant	HBB variant	HBD variant	Pathogenicity of variants	Predicted phenotype
HFY04	75.8	24	2.6	UK	(αα)^JX^/αα	ND	ND	P	α-TT
HFY15	78.3	25.3	2.2	1.4	(αα)^JX^/αα	ND	ND	P	α-TT
HFY28	69	21.1	2.2	UK	--^11.1^/αα	ND	ND	P	α-TT
HFY39	70.5	22.1	2.8	0.6	ND	Hb Lepore/β	ND	P	β-TT
HFY07	78.2	26	2.5	UK	ND	HBB:c.315+308delA Hete	ND	VUS	-
HFY21	75.6	23.4	2.1	UK	ND	HBB:c.315+308delA Hete	ND	VUS	-
HFY47	79.7	26.1	2.7	UK	ND	HBB:c.315+337A>G Hete	ND	VUS	-
HFY22	79.1	24.9	1.4	UK	ND	ND	HBD:c.127T>C Hete	P	δ

UK, unknown; ND, not detected; P, pathogenic; VUS, variants of unknown significance; α-TT, α-thalassemia trait; β-TT, β-thalassemia trait.

### 3.2 CATSA sequencing for subjects with α^0^ thalassemia variants

**FIGURE 3:**
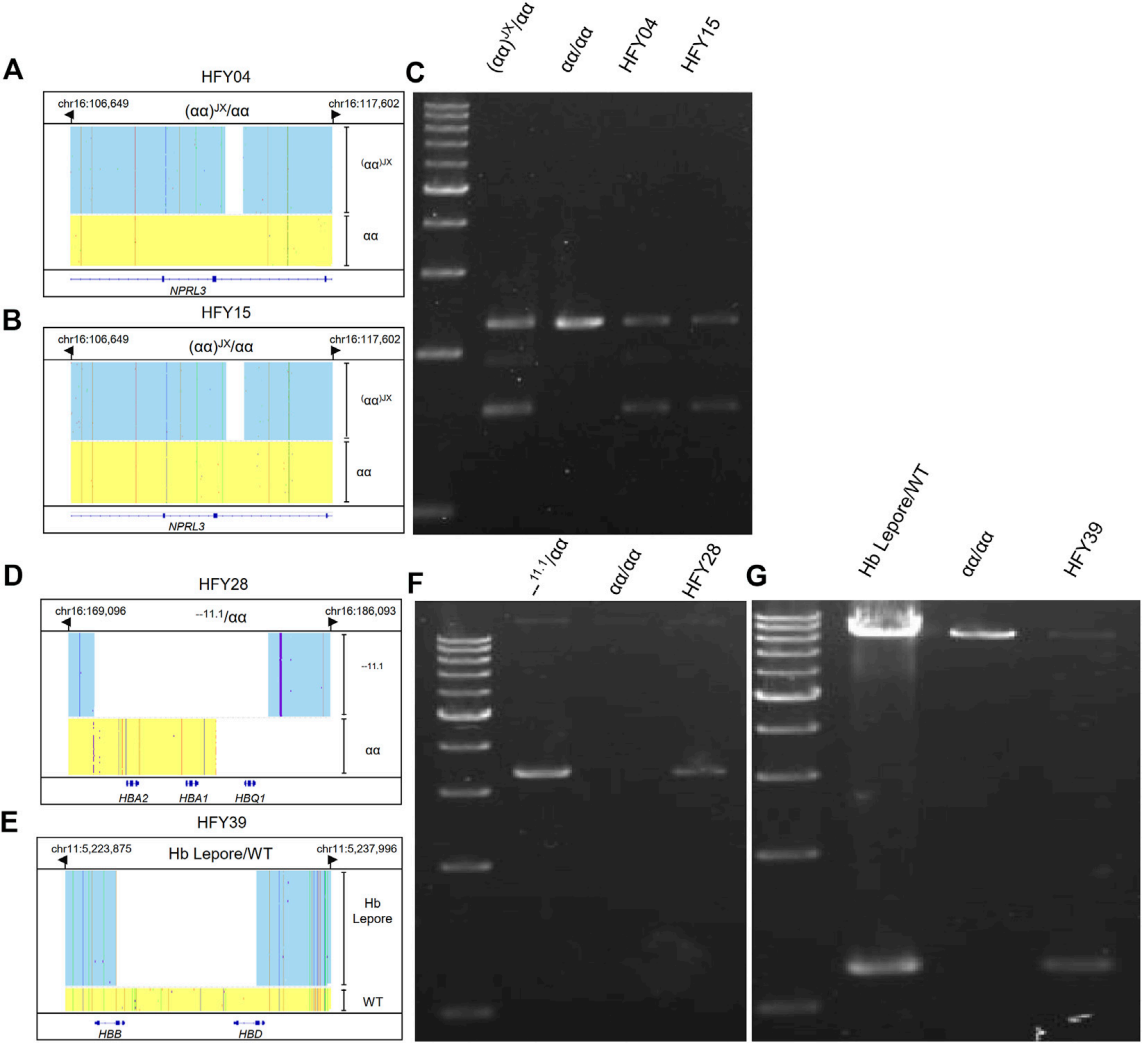
Integrative Genomics Viewer plots and PCR confirmation of the large deletions identified by CATSA. **(A,B,D,E)** IGV plots of (αα)^JX^/αα, --^11.1^/αα, and Hb Lepore/WT. Blue and yellow areas indicate two chromosomes. **(C,F,G)** GAP-PCR and agarose gel electrophoresis identified the three large deletions of (αα)^JX^/αα, --^11.1^/αα, and Hb Lepore/WT.

Both subjects HFY04 and HFY15 exhibited hypochromic microcytosis. HFY15 also had low Hb A2 of only 2.2%. CATSA revealed that both of them carried (αα)^JX^/αα, which was consistent with the hematological phenotype (Figures 3A,B). GAP-PCR specially designed for (αα)^JX^ confirmed both of them, indeed, harbored a heterozygous (αα)^JX^ variant (Figure 3C). (αα)^JX^ is a rare 742-bp deletion at the HS40 region, which is a remote regulatory element upstream of the α-globin genes and can bind to multiple transcription factors. There are four remote regulatory elements for the α-globin genes and HS40 appears to be the major regulatory element. HS40 deletion can impact the expression of *HBA1* and *HBA2* and cause α^0^ variants. Subject HFY28 not only suffered from hypochromic microcytosis but also had low Hb A2 (2.2%). CATSA found he presented --^11.1^/αα, which removes the entire *HBA1* and *HBA2* and also causes α^0^ variants (Figure 3D). Similarly, GAP-PCR specially designed for --^11.1^ confirmed he, indeed, harbored a heterozygous --^11.1^ variant (Figure 3F).

### 3.3 CATSA sequencing for subjects with thalassemic variant Hb Lepore

Subject HFY39 had an MCV of 70.5 fl, MCH of 22.1 pg, Hb A2 of 2.8%, Hb F of 0.6%, and abnormal hemoglobin of 9.3%, while CATSA identified a heterozygous Hb Lepore variant (HGVS: NG_000007.3: g.63632-71046del) resulting in the deletion of approximately 7.4 kb of DNA (Figure 3E). Hb Lepore carriers exhibit a β-thalassemia trait phenotype with microcytosis and hypochromia, which is consistent with subject HFY39’s phenotype. GAP-PCR specially designed for Hb Lepore confirmed he presented a Hb Lepore variant (Figure 3G). It is the first time that Hb Lepore was identified in Hunan Province. There are 5 subtypes of Hb Lepore: Hb Lepore-Boston-Washington, Hb Lepore-Baltimore, Hb Lepore-Hollandia, Hb Lepore-Leiden, and Hb Lepore-ARUP. The Hb Lepore identified in this subject belongs to Hb Lepore-Boston-Washington, which is the most commonly reported subtype observed worldwide and in many ethnic groups but is more frequent in Mediterranean countries.

### 3.4 CATSA sequencing for subjects with hemoglobin variants of potential pathogenicity

**FIGURE 4:**
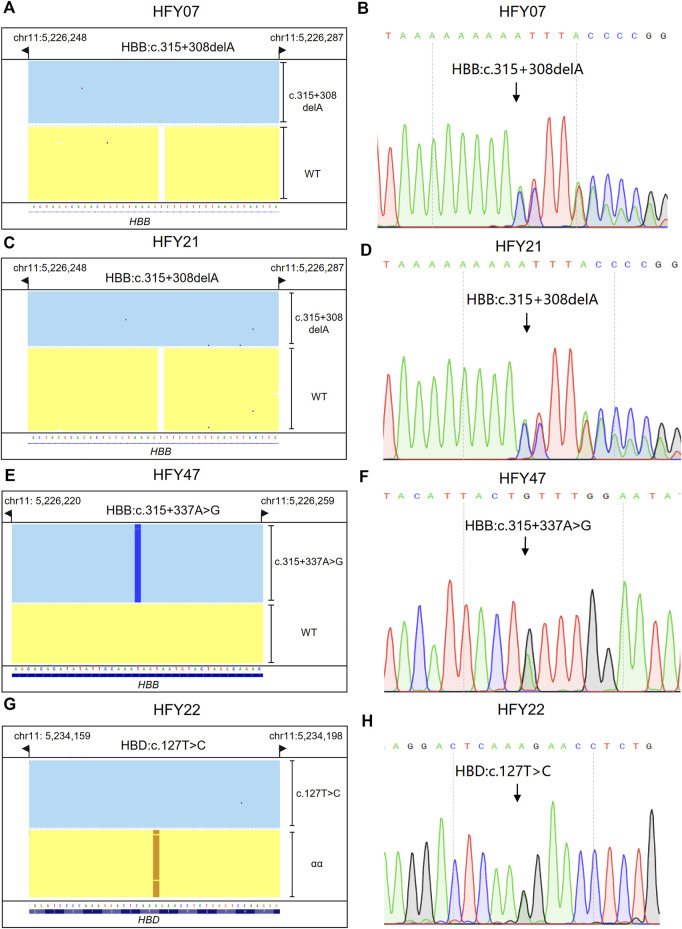
Integrative Genomics Viewer plots and Sanger sequencing of the SNVs and indels identified by CATSA. **(A,C,E,G)** IGV plots of HBB:c.315+308delA heterozygote, HBB:c.315+337 A>G heterozygote, and HBD:c.127T>C heterozygote. Blue and yellow areas indicate two chromosomes. **(B,D,F,H)** The SNVs and indels of HBB:c.315+308delA heterozygote, HBB:c.315+337 A>G heterozygote, and HBD:c.127 T>C heterozygote identified by Sanger sequencing.

Subjects HFY07, HFY21, and HFY47 had no thalassemia variants but other SNVs detected in their *HBB* genes by CATSA; HFY07 and HFY21 harbored HBB:c.315+308delA heterozygote; and HFY47 harbored HBB:c.315+337A>G heterozygote (Figures 4A,C,E). Sanger sequencing verified the results of CATSA for these subjects (Figures 4B,D,F). Since these subjects all had hypochromic microcytosis, these SNVs presented potential pathogenicity of thalassemia.

### 3.5 CATSA sequencing for subjects with HBD variants

Subject HFY22 was a 27-year-old woman with a large amount of menstruation, lasting about 7–10 days. Ultrasound examination indicated that there is a uterine adenomyoma, and she was intermittently taking iron supplements. She suffered from low MCV, MCH, and Hb A2. CATSA found she presented HBD:c.127T>C heterozygote, which is located in exon 2 of *HBD* (Figure 4G). Sanger sequencing also verified the results of CATSA for this subject (Figure 4H). This variant affects the stability of δ-globin, resulting in a decrease in Hb A2 (Liu et al., 2013).

## 4 Discussion

In this study, CATSA was conducted for the subjects with no thalassemia variants identified by routine PCR, Sanger sequencing, and specially designed GAP-PCR. In the 49 subjects tested by CATSA, eight subjects had variants identified. Independent experiments confirmed the results of CATSA were all correct. CATSA showed great advantages compared to the other genetic tests, which all had false-negative results.

Among the eight subjects with variants identified by CATSA, one subject presented a Hb Lepore variant. Hb Lepore carriers exhibit a β-thalassemia trait phenotype with microcytosis and hypochromia, which result from the decreased transcription of the δβ-hybrid chain. In the United Kingdom, the Antenatal Screening Program guidelines require genetic testing for Hb Lepore and providing genetic counseling to couples at risk of having a child affected with a clinically significant form of thalassemia (Guo et al., 2015). However, Hb Lepore is not included in the routine carrier screening of thalassemia in China. Since missed detection of this variant may result in inaccurate prenatal diagnosis and thus lead to the birth of children with thalassemia, it is important to include this variant in the carrier screening of thalassemia in China. Similarly, it is also crucial to add (αα)^JX^ and --^11.1^ in the carrier screening. Variants in *HBD* will not cause thalassemia, but they can lead to the decrease in Hb A2. The elevation of Hb A2 caused by β-thalassemia may be impaired by simultaneous HBD variants. Therefore, it is important to include HBD as well.

Among the eight subjects with variants identified by CATSA, two subjects harbored HBB:c.315+308delA heterozygote. Previous research studies showed that four cases with this variant exhibited mild decreased MCV and MCH levels. However, one additional case reported in a 27-year-old Chinese woman showed no clinical presentation (Hb 10.6 g/dl, MCV 79.1 fL, MCH 26.0 pg, Hb A 97.4%, and Hb A2 2.6%) (https://www.ithanet.eu/db/ithagenes?ithaID=3683). In this study, CATSA also found that two subjects with this variant suffered from hypochromic microcytosis. It seems that HBB:c.315+308delA is not a benign variant but a pathogenic variant. In order to determine whether it is a real pathogenic variant for thalassemia or not, more cases for this variant need to be accumulated.

Nonetheless, there are certain limitations in the present study. First, the current method only sequenced *HBA*, *HBB*, and *HBD* genes. There are still samples with no variants identified by CATSA. In the future, it is technically possible to cover other clinically significant regions, such as *HBG* and modifier genes. In addition, this study only compared CATSA with routine PCR, Sanger sequencing, and specially designed GAP-PCR. Future experiments can be conducted to compare CATSA with other frequently used methods such as multiplex ligation-dependent probe amplification (MLPA) and real-time PCR-based multicolor melting curve analysis (MMCA).

In conclusion, CATSA showed great advantages compared to the other genetic testing for carrier screening of thalassemia. In addition, previous research works also demonstrated that it is also highly beneficial for genetic analysis of other diseases, such as congenital adrenal hyperplasia and spinal muscular atrophy. Although the cost of CATSA is higher than that of the other methods, it is believed that as sequencing throughput increases and the cost decreases, CATSA will be widely applied for carrier screening of thalassemia and other genetic diseases.

## Data Availability

The data presented in the study are deposited in the SRA database of National Center for Biotechnology Information, accession number PRJNA909754. (https://www.ncbi.nlm.nih.gov/sra/PRJNA909754).
